# Supplementary material for: ‘We cannot just keep it in our palm’: A policy analysis of the integration of the case management of neglected tropical diseases into the health system of Liberia
Source: PLOS Glob Public Health. 2025 Dec 10;5(12):e0004328. doi: 10.1371/journal.pgph.0004328 (PMC12694817; doi:10.1371/journal.pgph.0004328)
Supplement: S1 Checklist — (DOCX) [file pgph.0004328.s001.docx]

Inclusivity in global research

PLOS’ policy on inclusivity in global research aims to improve transparency in the reporting of research performed outside of researchers’ own country or community and ensures that PLOS publications reporting global research adhere to high standards for research ethics and authorship. Authors of relevant research articles may be asked to complete the questionnaire below, which outlines ethical, cultural, and scientific considerations specific to inclusivity in global research. This questionnaire may be requested when researchers have travelled to a different country to conduct research, if research uses samples collected in another country, research with Indigenous populations or their lands, or if research is on cultural artefacts. Researchers travelling to another country solely to use laboratory equipment will not normally be required to complete the questionnaire. However, the questionnaire can be requested at the journal’s discretion for any submission – if you have been requested to complete this questionnaire by the PLOS journal you submitted to, please do so.

Please complete the questionnaire below and include this as a Supporting Information file with your manuscript. Note that if your paper is accepted for publication, this checklist will be published with your article in the supporting information files. Please ensure that you reference the checklist in the main body of your manuscript. We suggest adding a subsection ‘Inclusivity in global research’ to your Methods section and adding the following sentence: “Additional information regarding the ethical, cultural, and scientific considerations specific to inclusivity in global research is included in the Supporting Information (SX Checklist)”

The questions have been designed to be applicable to a wide range of study types, and there are subsections for both human subjects research and non-human subjects research. If any of the questions are not relevant to your research please mark them as “N/A” as appropriate.

**Ethical considerations, permits and authorship**

*This section is applicable to all research types.*

Provide details as to who granted permissions and/or consent for the study to take place in the Methods section of your manuscript. This should include the names of **all** ethics boards, governmental organizations, community leaders or other bodies that provided approval for the study. If individuals provided approval refer to these people by their role or title but do not list their name(s).

Reported on page number: 13

If there were any deviations from the study protocol after approval was obtained please provide details of these changes in the Methods section of your manuscript.
Did this study involve local collaborators that are residents of the country where the research was conducted or members of the community studied? If you do not have any authors from said communities, please provide an explanation for this below.

Reported on page number: N/A

Yes, local collaborators included several policy makers, technical and clinical staff from the NTD Programme along with Liberians working based in Liberia working for an International NGO. The collaborators are all listed as co-authors in the paper.

Everyone listed as an author should meet PLOS’ criteria for authorship and all individuals who meet these criteria should be included in the author byline, rather than the acknowledgements. For further information please see the journal’s Authorship Policy.

**Human subjects research (e.g. health research, medical research, cross-cultural psychology)**

Did you obtain written informed consent from a representative of the local community or region before the research took place? How did you establish who speaks for the community? Details of written informed consent obtained from study participants should be reported separately in the Methods section of your manuscript.

Formal written consent was obtained from the Ministry of Health’s National NTD Programme in Liberia, which was identified as the appropriate authority responsible for overseeing NTD policy and programme implementation. This approval followed extensive consultation as part of the NIHR-funded REDRESS research programme.

In addition, ethical approval was obtained from the Institutional Review Board of the University of Liberia (ACRE IRB #23-01-356) and the Liverpool School of Tropical Medicine (REC #22-056). The legitimacy of institutional stakeholders was established through a participatory stakeholder analysis conducted in 2020, which included national, regional and county level actors. This analysis helped to identify relevant decision-makers and informed both the research design and the purposive selection of interview participants.

Written informed consent was obtained from all individuals who participated in key informant interviews. Participants received an information sheet and consent form at least 24 hours in advance, and consent was reaffirmed at the start of each interview. All interviews were conducted with careful attention to participant comfort, confidentiality, and voluntary participation

How did members of the local community provide input on the aims of the research investigation, its methodology, and its anticipated outcome(s)?

Input from local stakeholders—including representatives from the Ministry of Health, county health teams, and persons affected by NTDs—was integrated into the research design through a participatory stakeholder analysis conducted as part of the REDRESS programme in 2020. This analysis involved national and sub-national actors as well as individuals with lived experience of NTDs. It informed the identification of priority stakeholders, the focus of the research questions, and the methodological emphasis on systems-level and county-level engagement.

While the study did not involve community-level intervention, the stakeholder analysis prioritised community and county-level voices, particularly County NTD focal points, whose roles were previously underexplored. Their input influenced the decision to adapt the data collection strategy and interview guides to better capture their perspectives and experiences.

Throughout the research, ongoing collaboration with the National NTD Programme ensured alignment with country priorities and policy relevance. Preliminary findings were shared with stakeholders for validation and feedback, and the dissemination strategy includes sharing results in accessible formats with local and national partners.

When engaging with the local community, how did you ensure that the informed consent documents and other materials could be understood by local stakeholders?

The consent form was drafted and reviewed by colleagues in Liberia to ensure it was understable and appropriate to the context. The form was shared at least 24 hours before interviews and significant time was allotted to the interview window to review and discuss all the information that was included in the informed consent dociments and other materials.

Will the findings of the research be made available in an understandable format to stakeholders in the community where the study was conducted (e.g. via a presentation, summary report, copies of publications, etc.)? Please provide details of how this will be achieved.

The findings were presented to participants at the REDRESS Dissemination workshop in 2024 and an additional dissemination activity in February 2025. The presentation was interactive and the opportunity was given to ask questions and give feedback. Additionally, the quotes used in the manuscript were checked by participants within Liberia to ensure they were happy with them being included in a publication, this resulted in the removal of two quotes at the request of participants who were concerned they may be identifiable.

**Non-human subjects research using specimens/ animals collected as part of the study, or those housed in archival collections. Examples include archaeology, paleontology, botany and zoology.**

Did the permission you obtained from a local authority to perform the study include an agreement on access to outputs and benefit sharing? This may include procedures to enable fair distribution of the benefits and resources arising from the research performed. Please include any details of Prior Informed Consent and Benefit Sharing Agreements obtained. These may be required by field-specific regulations, for example the Convention on Biological Diversity (CBD) and the associated Nagoya Protocol.

If the material used in your study was imported, please A) provide the year it was imported and B) indicate whether permits were obtained to import/export the materials used, C) provide details of any permits obtained. If this information is not available, please indicate this.

If you used archival specimens, please state how the material used in your study was acquired by the institute it is held in and provide details of any permits obtained for the original excavations/ sample collection. If this information is not available, please indicate this.

How was the potential cultural significance of the materials collected in your study to local communities considered in your research design? Were Indigenous peoples and/or local researchers and institutions involved with archaeological excavations / collection of specimens? If so, please provide a description of their involvement.

If your manuscript includes photographs of human remains please indicate whether authors obtained permission from descendants or affiliated cultural communities to do so.
